# The “Ineffable Freemasonry of Sex”: Feminist Surgeons and the Establishment of Radiotherapy in Early Twentieth-Century Britain

**DOI:** 10.1353/bhm.2007.0008

**Published:** 2007

**Authors:** Ornella Moscucci

**Keywords:** radiotherapy, radical abdominal hysterectomy, women surgeons, cervical cancer, Marie Curie Hospital

## Abstract

In 1924 the London Committee of the Medical Women’s Federation was instrumental in establishing a clinic for the purpose of investigating the radium treatment of cervical cancer. The scheme was later to evolve into a hospital, the Marie Curie, where adherence to the methods developed in Stockholm served to establish radiotherapy as an alternative to surgery in cancer of the cervix. This article examines the women’s contribution in the light of feminist and professional struggles over the relative merits of surgery and radiotherapy. It argues that radiotherapy was an issue of special interest to women surgeons, not only because of the long history of feminist opposition to gynecological surgery, but also because it could widen women’s access to the medical profession in the face of male exclusion from training posts and honorary appointments at voluntary hospitals.

## Introduction

Historians have recognized the part played by medical women in the establishment of radiotherapy in Britain.^1^ The Marie Curie Hospital, the first special hospital for the radium treatment of cancer, grew out of a clinic established in 1924 by the London Committee of the Medical Women’s Federation (MWF). Staffed entirely by medical women, the institution became famous for its outstanding success with the radium treatment of cervical cancer at a time when radical surgery was still regarded as the mainstay of treatment in “operable” cases. By the late 1930s the hospital had expanded its work to include rectal and breast cancer. It treated some seven hundred in-patients annually in thirty-nine beds, with facilities for radium and X-ray therapy, hostel accommodation for out-of-town patients, and up-to-date pathological and research laboratories.

In this article I reexamine the women’s contribution in the light of the debate over the relative merits of surgery and radiotherapy. Central to this story was the contemporary concern with cervical cancer. The leading cause of female cancer death in Britain between 1840 and 1940, it had been the focus of therapeutic intervention since the last quarter of the nineteenth century with the development of both vaginal and radical abdominal hysterectomy. The latter, a procedure usually associated with the name of Ernst Wertheim, was by 1920 the treatment of choice for cervical cancer, despite widespread public and medical anxiety about its high mortality and “mutilating” consequences. It was partly because cervical cancer was an exclusively female disease, partly because of the long history of feminist opposition to gynecological surgery, I argue, that radiotherapy for cervical cancer became an issue of special interest to women surgeons, many of whom were active in the suffrage movement and in various campaigns to improve women’s health. In addition, radiotherapy offered medical women career opportunities that were not readily available within the male-dominated field of surgical practice. A marginal specialty, it could accommodate female outsiders in the face of male exclusion from training posts and honorary appointments at major voluntary hospitals. Gender politics, in other words, were a significant dimension of the debate over the value of radiotherapy for cervical cancer. Exploring women’s contribution is thus important in order fully to [Other P-139] appreciate the broader political and social forces that shaped cancer care in early twentieth-century Britain.

## Surgeons Ascendant

The leading cause of cancer death among British women until 1940, for much of the nineteenth century century cervical cancer inspired dread in doctors and patients alike. When John Williams, professor of midwifery at University College London, delivered the Harveian Lectures for 1886, he stated that no apology was needed for making this disease the subject of his talks: “The frequency with which it is met, its irresistible progress, the horrible sufferings which it entails upon its victims, the utter helplessness of medicine in its presence, and its fatal character, all alike join in demanding a careful study of its insidious onset, and destructive habits.”^2^ In Alban Berg’s 1917 opera *Woyzeck* the subject of cancer of the womb was introduced by a short musical sequence constructed around the note B, which came to symbolize death throughout the opera.^3^

Attempts to treat cervical cancer by amputation and hysterectomy were made at the beginning of the nineteenth century, but they rarely eradicated the disease, and the fearful, often fatal hemorrhages that attended the treatment served to discourage further attempts. As the obstetrician Charles West remarked in 1858, “the supposed triumphs of surgery in cutting short the disease . . . were, for the most part, purely imaginary; and the trophies once displayed in our museums are now generally put out of sight, as the mementoes of a pathological blunder and a needless operation.”^4^ Cauterization in the “early” cases (i.e., where the cancer did not extend beyond the limits of the uterus) and palliation for the more advanced were thus standard practice in Britain for much of the nineteenth century.

In the last quarter of the century, however, the pendulum began to swing the other way and surgeons began to take a decidedly more interventionist approach to the disease. Amputation of the cervix was reintroduced for the early cases, while the development of abdominal surgery made it possible to tackle the more advanced cases by extirpating the uterus. The first attempts in this direction were made in the late 1870s [Other P-140] by Wilhelm Freund, a German gynecologist who pioneered a procedure involving the removal of the uterus and parametrium. The purpose of Freund’s hysterectomy was twofold: first, to root out the disease by removing not only the cancerous tissue, but also a good margin of apparently healthy tissue (hence the term “radical operation”); second, to extend the field of operability. But the dreadful mortality that attended Freund’s abdominal hysterectomy (70 percent) caused widespread opposition, leading to the development of a vaginal procedure attended by a lower mortality (about 10 percent on average by 1889).^5^ A further evolution of the method involved the introduction of episiotomy to widen the vagina so as to facilitate the excision of a larger portion of the parametrium, and the isolation of the ureters. The Viennese gynecologist Friedrich Schauta used this technique in 1901 to develop his own method, which involved the removal of a cuff of vagina together with the uterus.

During the 1890s, the belief that cervical cancer spread centrifugally along the lymph nodes provided the impetus for the development of radical abdominal hysterectomy. The model for this procedure was the radical mastectomy operation popularized by William Halsted in the early 1890s. Halsted advocated the removal of the breast, the larger of the two chest wall muscles, and the axillary glands in one piece, to prevent both the dissemination of cancer cells and local recurrence.^6^ Reasoning by analogy, some surgeons and gynecologists criticized vaginal hysterectomy on the grounds that it could not deal with the problem of lymphatic spread. They argued that only the removal of the pelvic nodes, as well as the ovaries, the fallopian tubes, the uterus, and the healthy tissue around it could prevent recurrences. This extensive surgery is not viable unless an abdominal operation is performed. It is far easier to remove the upper portion of the broad ligaments with the fallopian tubes and ovaries in abdominal hysterectomy than when the operation is performed from below. Furthermore, the operation permits the removal of the lymph nodes lying on the iliac vessels, which is impossible by the vaginal route.

Radical abdominal hysterectomy was a German-American innovation: German from Freund who originated the abdominal route, and American [Other P-141] from the surgeons who developed it. In 1895 Emil Ries of Chicago was the first to show, by experimenting on dogs and cadavers, that a radical operation on the lines of Halsted’s mastectomy was feasible; and in the same year John G. Clark, a colleague of Halsted’s at Johns Hopkins Hospital, performed it on a living woman.^7^ He was followed by Howard Kelly, also of Johns Hopkins, who perfected the method of dissecting the ureters from the region around the growth. Clark subsequently reported twelve cases of radical abdominal hysterectomy, though the technique used in each varied slightly: in some he had removed the lymph nodes, in others the parametrium and part of the vagina.

Total abdominal hysterectomy was subsequently standardized and popularized by the Austrian gynecologist Ernst Wertheim. A student of the legendary pioneer of abdominal surgery Thomas Billroth, Wertheim worked with the gynecologist R. Chobrak in Vienna and in 1891 he became first assistant to Friedrich Schauta. When he was appointed head of the Department of Gynecology at the Bettina Pavilion of the Elisabeth Hospital in Vienna, Wertheim obtained his own operating facilities and he then began to develop an extended abdominal operation for the treatment of cervical cancer. Wertheim’s procedure was essentially a modification of the abdominal hysterectomy devised by Ries and Clark. Its distinguishing features were the thorough removal of the cellular tissue around the uterus, and the clamping of the vagina beneath the cancer (this was aimed at isolating the growth before removal, so as to avoid the risk of “infecting” the healthy portion of the vagina with cancer cells). Wertheim did not initially remove the lymph nodes as a routine; however, he subsequently became convinced that lymphatic involvement was a feature in the majority of cases, so he extended the procedure to include the pelvic glands.^8^

Wertheim claimed that his chief motive was to offer hope to those women who were “shut out from life” because of advanced carcinoma, but the high mortality from the operation was a cause for concern to many of his contemporaries. The fiercest criticisms came from feminists, who [Other P-142] linked the development of “mutilating” operations with male violence, the uncaring attitudes of practitioners, and the use of animals in laboratories. Many feared that indifference to the sufferings of animals would encourage a loss of humanity: the suspicion was that it could be animals first, and women and workers next.^9^ But gynecologists themselves had their doubts about radical abdominal hysterectomy. A British critic, the gynecologist Frederick McCann, remarked in 1907: “unless temporary or permanent benefit can be promised to the patient, it is not justifiable to subject her to a prolonged and dangerous operation which cannot completely remove the disease, more especially as the palliative operations and methods of treatment give considerable relief in the advanced stages of the disease and are less dangerous.”^10^ McCann was echoed by William Japp Sinclair, who roundly condemned the extended radical abdominal hysterectomies as “homicidal vivisections, which nothing hitherto advanced in their support appears to palliate, much less to justify.”^11^ Similar concerns were voiced in France and the United States.^12^

Such views need to be placed in the context of the evolution of British gynecology from about 1800 onward. Throughout the nineteenth century the specialty was dominated by practitioners who aspired to be gentlemanly physicians and spurned surgery as a lowly occupation, beneath the dignity of educated medical men. By the end of the century, however, the status of surgery was on the rise, and the younger men and women who were entering the profession had fewer prejudices against operative treatment. At the same time, the process of specialization was attracting surgeons to the field of gynecology, bringing to an end the conservative phase that had dominated its practice since the late eighteenth century. Wertheim’s operation was caught in the crossfire between the older generation of obstetric physicians on the one hand, and the younger generation of surgeons and obstetricians with surgical aspirations on the other.

The turning point in the fortunes of the extended abdominal hysterectomy in Britain came in 1905, when Wertheim introduced his method at an epoch-making meeting of the British Medical Association. At this [Other P-143] meeting the eminent surgeon impressed his audience with claims that 30 percent of the cases treated by his procedure were free from recurrence after five years, as against the 10 percent or less obtained by simple abdominal or vaginal hysterectomy. Furthermore, he asserted that his operation was applicable to just over 50 percent of all cases of carcinoma of the cervix, instead of the 10–15 percent that was the limit of the lesser procedures. Wertheim had had thirty deaths in his first one hundred cases, but by the time he read his paper the operative mortality had fallen to 7 percent.^13^

Wertheim’s operation was pioneered in Britain by abdominal surgeons: Cuthbert Lockyer of London’s Samaritan Free Hospital, and Victor Bonney and Comyns Berkeley of the Middlesex Hospital. They were later joined by obstetricians like William Fletcher Shaw of Manchester, who founded the Royal College of Obstetricians and Gynaecologists in 1929. Bonney and Fletcher Shaw in particular continued to champion Wertheim’s method during the 1920s even as the new techniques of radiotherapy challenged the dominance of surgeons in cancer therapy. Bonney saw surgery as the key to the rise in status of gynecology. A stalwart supporter of the Royal College of Surgeons, he wanted to maintain gynecology within the broader sphere of surgery, and he was determined that radiotherapy should remain the handmaiden of surgery.^14^ But it should not be assumed that Bonney was insensitive toward women’s feelings about gynecological surgery—indeed, one of his main claims to fame was the development of a conservative operation for uterine fibroids (myomectomy) at a time when hysterectomy was the standard treatment for the condition. “Apart from its physical value,” he once commented, “the womb has for most women a sentimental value which, however illogical, cannot be lightly dismissed.”^15^ Bonney’s views may have been influenced by events in his private life: in 1905 his wife had a hysterectomy for fibroids, and the couple was childless.

## New Weapons, New Hopes

Although radical abdominal hysterectomy was “running the gauntlet of an animated professional criticism” in the early 1900s, few gynecologists [Other P-144] questioned the propriety of surgical treatment.^16^ “That cancer of the uterus is a hopeless and uniformly fatal disease is a proposition that has been true in the past through the whole period of human history during which the disease has been known,” wrote the obstetrician Arthur Lewers in 1902:

But the position is now entirely altered, since we now know that, if only cases are recognised in an early stage, a fair proportion may be permanently relieved by operation. . . . Hence it may be hoped that in the future suspicious symptoms will lead to prompt and thorough investigation, since at all events a diagnosis of cancer of the uterus in an early stage is now by no means equivalent to the diagnosis of a fatal disease.^17^

The problem for gynecologists was that many patients presented with disease that was too far advanced for operative treatment. Thus when X rays and radium were discovered, hopes were immediately raised that irradiation techniques might be beneficial in the “inoperable” cases. It was the apparent success of X rays and radium with both benign and malignant skin conditions that led to the application of the healing rays to less accessible tumors in the body’s natural cavities: the mouth, the nose, the throat, the rectum, and the uterus. In 1903 Pierre Curie suggested that the advantage of radium over X rays was that it could be applied accurately to the place requiring treatment when contained in a fine tube.^18^ This brought radium into the range of surgical treatments, attracting the attention of surgeons. By October 1903, a design for an aluminum tube to enable the insertion of radium into a tumor had appeared in the *Archives of the Roentgen Rays*, the first British radiological journal.^19^

While attracting widespread medical and public interest, the use of various sources of radiation in the treatment of cancer was initially viewed with suspicion by the medical profession. X-ray therapy was associated with the fringe practice of medical electricity, while radium therapy had its roots in heliotherapy, spa treatments, and the use of cauteries. The removal of cancer by cauterization was one of the oldest procedures in medicine, but by the early 1900s surgery had become the treatment of choice and any therapy that challenged the orthodoxy was branded as quackery.^20^

In Britain the first reports of X-ray and radium therapy for gynecological disease began to appear in the early 1900s. In 1904 the *Journal of Obstetrics and Gynaecology* (founded in 1902) first described attempts to treat cancer of the vagina and of the breast by X-ray therapy, but the results were said to be disappointing.^21^ In 1905, the obstetrician John Shields Fairbairn commented: “so far, nothing has been proved of the value of the rays as a therapeutic measure.”^22^ In the second edition of W. Playfair and T. Allbutt’s *System of Gynaecology*, published in 1906, the gynecologist Amand Routh, of London’s Charing Cross Hospital, wrote of testing the effects of radium in a case of inoperable cancer of the cervix: he had observed a marked reduction in symptoms, but the growth had continued to spread in the deeper tissues.^23^

By 1913 there was mounting evidence of radium’s palliative effects in inoperable cases. In the first report of the London Radium Institute, founded in 1911 at the instigation of King Edward VII, it was stated that a total of thirty-nine cases of cancer of the uterus had been treated during the first year of activity of the institution: three patients had been discharged apparently cured, nineteen were “improved.” Hayward Pinch, the medical superintendent, remarked that “in cases of inoperable malignant disease in this situation radium will often bring about results which cannot be attained by any other known method of treatment. . . . The rate of growth is checked, sometimes completely arrested, and the surrounding infiltration and induration are so much lessened that in a few instances cases previously declared to be inoperable become operable”; he noted, however, that the treatment was rarely curative: “though it may, and often does, check the rate of growth, yet in most cases dissemination will sooner or later occur, and the disease spread to parts beyond the effective range of radium.”^24^ Leading surgeons sought to damp down expectations. The rapid rise and demise of wonder cures such as Robert Koch’s much-hyped tuberculin remedy twenty years earlier engendered an attitude of caution. As the surgeon Henry T. Butlin observed in 1909, “Berlin did a fine business while the craze lasted, but many of the patients spent more than they could afford to do on a treatment which was purely experimental, while [Other P-146] others died miserably in hotels and lodging-houses”; British practitioners should not send patients to Paris for radium treatment merely in the hope that it might “do some good.”^25^

Gynecologists’ enthusiasm for X rays and radium therapy nonetheless rose during the 1910s, stimulated by the growing realization that radical cancer surgery had reached its limit. “The last card in the operative treatment of malignant disease appears to have been played,” wrote Victor Bonney in 1915: “The hope that in the future more searching and safer means of cure than the scalpel and the dissecting forceps may be discovered is growing brighter.”^26^ Radiotherapy at first found a place in the treatment of inoperable cases, both as a palliative and as a means of extending the field of operability. After the First World War, surgeons began to use radium and X rays postoperatively in an attempt to diminish the tendency to recurrence.

A major change in philosophy was evident by the early 1920s, when advocates began to suggest that radiation alone could be used in operable cases. In 1920 the *British Medical Journal* argued that the treatment of cervical cancer by radium therapy should no longer be confined to inoperable cases: “More modern methods . . . together with a better appreciation of dosage and its effects, are undoubtedly pointing in the direction of radium therapy in earlier cases—even in those which are operable.”^27^ Advocates of Wertheim’s hysterectomy were unconvinced, but increasingly during the 1920s the achievements of surgery were challenged by radiologists and gynecologists themselves. Enthusiasm for irradiation among surgeons rose sharply after 1925, prompting talk of a “boom of radium.”^28^ A notable convert was Comyns Berkeley, Bonney’s associate at the Middlesex: he established a radium clinic at the Lambeth Hospital in London, and in the late 1920s he became a member both of the first National Radium Commission and of the League of Nations Commission on Radium.^29^

According to advocates, the main advantage of the new technique was its very low mortality compared with radical abdominal hysterectomy (approximately 2 percent in 1933, compared with an average death rate [Other P-147] of 15.3 percent for Wertheim’s operation).^30^ Furthermore, radiotherapy was said to reduce the amount of “mutilation” to a minimum. This was a key point for the champions of irradiation, since the fear of operation and mutilation was widely regarded as a major cause of delay in treatment. Radiation therapy had none of the disadvantages of surgery; hence, it could help reduce delay and boost sufferers’ chances of a cure.

From the point of view of the patient, the added attraction of radiotherapy was that it was less disruptive of ordinary daily activities than major abdominal surgery. One of the factors that contributed to the popularity of the Erlangen deep X-ray method in the 1920s was that the treatment could be completed in a short time and on an outpatient basis, thus enabling continuation of paid employment. When the gynecologist Louisa Martindale decided to adopt the Erlangen method in 1922, she found that the treatment appealed to many “doctors, headmistresses and other professional women as well as others who disliked to face an operation involving hospitalisation and a long convalescence, and—what to some is a serious matter—the loss of the uterus.”^31^

Treatment with X rays or radium was not an easy option, however. Exhaustion, severe anemia, nausea, and sterility were the norm after a course of treatment with the deep X-ray therapy favored by Martindale, until it was realized that the inhalation of the noxious gases produced by the apparatus was an important factor in the causation of X-ray sickness. Proper ventilation and the removal of the apparatus from the treatment-room helped reduce the side effects of the treatment, but by the 1930s the search for ever more powerful X-ray machines was provoking renewed anxiety about the method. Radium researchers and medical physicists themselves deplored the “gigantism” of the new high-voltage apparatus and the “subjugation” of the patient to the machine, arguing that in their desire for X rays of greater penetrating power, radiologists were losing sight of the most important principle of successful treatment: “Primum non nocere.”^32^

Meanwhile, radium therapy had become increasingly invasive with the development of surgical techniques aimed at opening the tumor to the [Other P-148] radium—the so-called surgery of access. Frans Daels’s intrapelvic method, for example, involved incisions through the pelvis and blunt dissections of tissues followed by the insertion of radium containers directly into the growth. “Radio-surgery” was a response to those surgeons who claimed that radiotherapy could not deal with the lymphatic glands, but the insertion of needles did increase the risk of septic infection, which was one of the most common causes of death after radiotherapy.^33^

## Turf Wars

As the new radiation techniques found a niche in cancer therapy, questions arose as to who should determine and carry out the treatment. The problem was usually formulated in terms of effectiveness and expertise, but the underlying issue was one of control. Who was to have jurisdiction over the cancer patient? In Britain, X-ray therapy was developed mostly by radiologists working in medical electricity departments, and gynecologists showed little interest in the technique. As Louisa Martindale noted in her autobiography, published in 1951, it was not surprising that in England the method had never been popular among the surgeons: “A surgeon is naturally anxious to treat the patient himself,” she wrote, “and it was not the British custom to equip gynaecological clinics with their own X-ray facilities.”^34^ Martindale was unusual in being one of the first gynecologists in Britain to use X rays in her own practice. A fluent speaker of German, in 1913 she heard about the promising results that Professors Bernhard Krönig and Karl Gauss were obtaining with radiation therapy and she went to Freiburg to learn the techniques; what she saw impressed her so much that on her return to England she invested in an X-ray machine and began to treat certain benign conditions of the uterus, and cancer of the breast. By the early 1920s she was experimenting with the Erlangen method, which she had seen demonstrated in Professor Ludwig Seitz’s department. Martindale’s view was that the clinician responsible for determining and carrying out the treatment should be the gynecologist rather than the radiotherapist. This was because of the gynecologist’s diagnostic expertise: “Careful and accurate diagnosis,” she observed in her autobiography, “is the main factor in obtaining success and, for this reason, it was held in Freiburg that the treatment should be carried out by the gynaecologist in an X-ray therapeutic department attached to a [Other P-149] gynaecological clinic.”^35^ Martindale realized that the high fees charged for gynecological operations provided a strong incentive to surgery. Anxious that financial considerations might bias her judgment, she resolved to adopt the policy already implemented at Freiburg and charge the same fee for a hysterectomy as for X-ray treatment.

The question of control in radium therapy was more complex than for X-ray treatment. Some radiologists used radium as well as X rays, but most radium therapy was carried out by surgeons, gynecologists, dermatologists, and laryngologists; most importantly, surgeons controlled the insertion of radium into tumors. When the first specialized radium institutes in Britain were established, tensions arose over the control of cases. How could referring practitioners and specialists protect their professional and financial interests? The solution adopted at the London Radium Institute was to set up a complex hierarchical structure of tasks and responsibilities aimed at reducing the impact of the institution on the private practice of referring doctors. As the chairman of the Institute’s medical committee, the eminent surgeon Sir Frederick Treves, explained in a letter to Hayward Pinch, it was desirable that he should limit “as far as possible” his responsibilities with regard to the patients admitted to the Institute: “It rests with you to indicate the specific treatment by radium to be employed . . . to see that the treatment by radium that is recommended is carried out. . . . For that treatment you are responsible.”^36^ Diagnosis was, however, the responsibility of the referring doctor: no patient, whether necessitous or well-to-do, was to be admitted without a certificate from the practitioner, who thus retained his or her right to determine the therapy. In difficult cases, a third party was to be called in. Necessitous cases were to be seen by a member of the Honorary Staff, but a paying patient’s own doctor could choose any consultant. If Pinch was invited to choose a consultant, the patient would have to pay a fee, no matter where the consultation took place. Furthermore, when the methods of treatment required specialized skill, as for example in the insertion of radium into the uterus, Pinch was to hand over the patient to the appropriate specialist. The surgical manipulations required in such cases were free of charge for gratuitous patients; in all other cases a separate fee was payable directly to the specialist. Commenting on these arrangements, the *Lancet* remarked: “Sir Frederick Treves’s letter is so simple and clear a document that it might well be framed and hung in the corridors of the institute, if only to convince everyone that [Other P-150] the institute cannot be made use of to put fees into the pockets of any specially selected members of the medical profession.”^37^

The radium-therapy boom of the 1920s prompted the first attempts to discourage the entry of “inexpert” practitioners into radium therapy. Mounting public concern about the safety of radiation lent weight to the argument that radiology should be placed in the hands of experienced workers.^38^ Radiologists and surgeons with a special interest in radiotherapy were united in condemning empiricism in radiotherapy: “We see surgeons and gynaecologists rushing across to Brussels and elsewhere and, after a week of observation, returning as experts, writing up a few cases, and imagining that they are advancing scientific progress,” wrote scornfully the radiologist A. E. Barclay in 1929; in Barclay’s view, expert knowledge of radium and X rays was of far greater importance than expert surgery (which he alleged was a “comparatively simple matter” in the surgery of access): “The expert who controls the treatment should be the man who has expert knowledge of the most potent weapons—i.e., radium and X rays. . . . Till we realise that it is surgery and not radiation treatment which should now be regarded as the refuge of the destitute we shall not progress.”^39^

At the same time, advocates of radiotherapy sought to rebut the criticisms leveled by those surgeons who professed skepticism about the value of radium. One of the most vocal critics was Victor Bonney: the eminent surgeon had no truck with those who argued that the “purely operative” treatment of cervical cancer could no longer compete with irradiation in term of success. In an article published in 1930, Bonney strongly deprecated

as altogether premature the appeals that have been made to the younger generations of gynaecological surgeons not to embark on the operative treatment of cancer of the cervix, but instead to take up radium therapy, the present estimate of whose value in this connexion is founded solely on figures from abroad. Not until the results of reliable workers in this country are available shall we be in a position to properly appraise its effects, for it does not follow that the same measure of success attending a method of treatment in one country is necessarily attained when it is carried out in another country.^40^

William Fletcher Shaw also expressed concern about the lack of British statistics. “It is,” he wrote in a letter published in the *British Medical Journal* for 1927, “surely not asking very much of British radiology to publish statistics on, at any rate, a five-year basis.”^41^ The Manchester gynecologist had been using radium applications to increase the field of operability since the early 1920s, but he drew the line at the suggestion that radiation therapy should become a substitute for surgical excision in operable cases.^42^

In order to rebut these criticisms and distance themselves from the bulk of surgeons who took up radium therapy, advocates deployed the rhetoric of science, arguing for the establishment of separate institutions, staffed by experienced workers, in which methods could be tried, tested, and standardized. According to the gynecologist Malcolm Donaldson, one of the British pioneers of radium therapy for cervical cancer, the number of cases of malignant disease admitted to the general hospitals was not large enough to support systematic research programs, and most of the cases were distributed among clinicians who had no interest in research anyway: “Clinical research needs a great number of beds and a special organization, which I maintain is not possible in any general hospital.”^43^ As well as centralization and specialization, Donaldson recommended the establishment of a hierarchical system of management in which teams of workers would be subordinate to a fully qualified director of research. A key medical concept in the interwar period, the notion of teamwork was seen as an antidote to the old style of competitive individualism in medical practice: “Competition may have its merits in many walks of life,” wrote Donaldson in 1933, “but co-ordinaton and co-operation are far more important to the advancement of medical science.”^44^

Yet the logic of centralization did not necessarily entail the subordination of the surgeon to the radiologist. It could be argued that surgeon and radiologist were equal partners in a relationship, since one could do [Other P-152] in one sphere what the other could not do. As the *British Medical Journal* commented in 1933, the surgery of access demanded specialized skills that were outside the radiologist’s sphere of expertise: “*At first sight* the impression is that we are dealing with an essentially simple procedure which demands no special surgical or gynaecological qualifications.”^45^ At the same time, one needed to realize that the surgery of access was “only a means to an end,” namely, the “efficient distribution of the radiations from the radio-active material in the containers”; this was a “physical problem of no small complexity,” which demanded specialist skill and expertise: as the techniques of radiotherapy advanced, no surgeon or gynecologist could hope to acquire the necessary know-how simply by attending a short course in “radium surgery.”^46^ There were thus strong arguments for teamwork in the delivery of radium therapy: “Co-ordination, co-operation, and permanency of specialist staff are certainly not least among the essential conditions for success,” the journal observed.^47^ The *Lancet* agreed that the development of the surgery of access pointed to one conclusion: “even in the new era the radiologist and surgeon must work together.”^48^ Taking the argument further, the journal argued for the unification of surgical and radiotherapeutic tasks: “most efficient of all will be the man who can combine surgical with radiological technique in his own repertory.”^49^

It is against this background that we must now consider the Medical Women’s Federation initiative. Originally established as a clinic in 1924, by 1929 the MWF’s scheme had evolved into a thirty-bed hospital entirely staffed by medical women. Its work was to play a key role in establishing radiotherapy for cervical cancer, demonstrating the value of a rational “scientific” approach to the new radiation therapies.

## The MWF’s Cancer Committee

The MWF’s research scheme was the brainchild of pathologist Dr. Helen Chambers, the first cancer researcher employed by the Medical Research Council (MRC) at the Middlesex Hospital Cancer Research Laboratories. Chambers’s links with the Middlesex and the MRC were significant. The Middlesex had the longest tradition of specialized cancer work, and the strongest tradition of radium research: it was the first hospital to appoint [Other P-153] a medical physicist, and the original beneficiary of the radium acquired in 1919 by the Medical Research Committee, the predecessor of the MRC, from surplus government stock.^50^ In the 1920s the MRC played a key role in promoting clinical research into the medical uses of radium. As David Cantor has shown, the chief objectives of this work were to overcome surgical resistance to radium therapy, and to discourage the entry of inexpert surgeons into the field.^51^ Chambers’s initiative served to further both of these objectives.

Chambers’s association with the Middlesex Hospital went back to 1908, when she won a newly founded scholarship in cancer research. Chambers, who already held a part-time appointment at the Royal Free Hospital as assistant pathologist, worked in collaboration with Sidney Russ, the physicist and radium expert who was later to become the first secretary of the MRC’s Radiology Committee. Between 1911 and 1913 they produced a series of articles on the biological effects of radium, which established Chambers’s reputation in cancer research.

In 1915 Chambers resigned her appointment at the Royal Free Hospital in order to become pathologist to the Endell Street Military Hospital in London. At the end of the war she was one of the first to receive the Order of the Commander of the British Empire, and when the Endell Street Hospital closed, she returned to full-time cancer research with Professor Russ at the Middlesex Hospital.^52^ Although her main line of research was the induction of cancer immunity, using in particular small doses of X rays, she was enthusiastic about the possibilities of radium therapy, especially in the treatment of cervical cancer. In February 1924 she gave an address on radium therapy to the monthly meeting of the London Association of the MWF, in which she drew attention to the value and shortage of radium. She suggested that a body of medical women might cooperate in a study of one specific aspect of cancer therapy; an exploratory committee was immediately formed, with Dr. Chambers and four gynecological surgeons as members. The committee included a number of practitioners who had been active as feminists, suffragists, and champions of women’s health: Miss Maud Chadburn, founder of the South London Hospital for Women; Lady Florence Barrett, later to become Dean of the London [Other P-154] School of Medicine for Women; Lady Grace Maud Briscoe, physician to the Shoreditch Maternity Centre; and Louisa Martindale, founder of the New Sussex Hospital for Women. Miss (later Dame) Louise McIlroy, the first professor of obstetrics and gynecology at the Royal Free Hospital, and Miss E. Bolton, surgeon at the Elizabeth Garrett Anderson, also joined the committee as co-opted members.^53^

As a first step, it was decided to investigate the effects of radium therapy in carcinoma of the uterus. The choice of this cancer was not accidental. As we have seen, evidence from foreign centers suggested that radiotherapy was a viable alternative to surgery in the treatment of cancer of the cervix, yet many British surgeons still doubted that radiation could supplant Wertheim’s hysterectomy. A well-designed clinical trial had the potential to generate reliable statistics about the value of radium therapy in British practice, thus providing ammunition against the stalwarts of operative treatment.

Quite apart from the potential utility of the research, there were special reasons why the investigation should have been of interest to the members of the Cancer Committee. Cervical cancer was undoubtedly an issue that medical women could claim as their own. For a start, the etiology of cervical cancer was bound up with two major women’s causes: the prevention of venereal disease, and the reduction of the risks of childbearing. Earlier in the century suffragists and women doctors like Louisa Martindale had claimed that carcinoma of the cervix was one of the consequences of gonorrheal infections brought home by promiscuous husbands.^54^ The supposed connection between venereal disease and cancer served to highlight the penalty that women paid for male depravity, providing an argument against the double standard of sexual morality and the “enslavement” of women within marriage. As the question of maternal mortality and morbidity climbed to the top of the political agenda in the 1920s, medical attention shifted onto obstetric injury as a cause of cervical cancer. High rates of maternal death raised questions about the standards of obstetric care, suggesting to feminists that society was failing to appreciate women’s key role as childbearers. The high incidence of cervical cancer reflected the general neglect of women’s social and biological functions, implicitly demonstrating the need for reforms aimed at improving the material conditions of women’s lives.^55^

The ideology of medical women’s mission to other women came into play with regard to the diagnosis of cervical cancer. Since the second half of the nineteenth century, the case for women doctors had rested largely on the claim that women could provide medical and surgical care that did not violate women’s modesty. Women’s aversion to intrusive medical examinations was widely blamed as a cause of delay in the treatment of cervical cancer, so this was clearly an area where women’s otherwise unmet health-care needs would be best served by medical women. In a wartime fund-raising pamphlet for the Marie Curie Hospital, writer Vita Sackville-West emphasized the special role that medical women could play in cancer care as friends and confidantes to their patients:

[The hospital] exists to minister to peculiarly feminine ailments; and no one but a woman can know what they mean. They are ailments, which touch her in the most instinctive, primitive recesses of her being. . . . But if she knows she can go to a hospital where she meets with nothing but the indefinable freemasonry of sex; meets only other women who, though doctors, are speaking the same intimate language as herself; women to whom no revelation is novel, even the most secret fears and shyness and atavistic complexes—then her reluctance [to seek medical advice] may be modified and the danger taken before it is too late.^56^

Sackville-West also drew attention to the value of radiotherapy as a humane, woman-friendly alternative to “mutilating” surgery, deftly exploiting the long history of public concern over the treatment of charity patients in public hospitals. During the nineteenth century, fictional exposés of hospital practice had helped generate the belief that charity patients were being utilized as human subjects for vivisectionist experiments, fuelling working-class distrust of hospitals and surgical practice.^57^ Sackville-West alluded to these anxieties in order to drum up support for the Marie Curie Hospital:

Fear of the surgeon’s knife is a serious deterrent, but the application of radium appears to suggest no alarm. It is easy to see the extreme importance of this factor. . . . It must be recalled that many of the patients come from the poorer quarters of London, patients to whom “the orspital” meant a bewildered and helpless dread, but who delivered themselves over to the care of *this* hospital the more willingly for the knowledge that their poor bodies were not going to be [Other P-156] carved up while they lay under the arc-lights unconscious and without defence. On the contrary, they were going to find it a time of comfort and relaxation such as they had never known before in a hard-working life.^58^

This emphasis on medical women’s mission to the poor should not be taken as implying that the MWF’s venture was untainted by considerations of professional success. Medical women had been quick to see that the new field of radiation therapy and medical electricity could enhance their employment opportunities at a time when de facto sex discrimination placed serious constraints on their careers. As radiologist Mary Magill observed in 1925, medical women could no longer afford to ignore any branch of medicine, least of all radiology:

The special hospitals, staffed entirely by women, need women radiologists to take charge of their X-ray departments; women practitioners look for women radiologists to whom they can send their patients; and women patients do, in the majority of cases, prefer that opaque meals and similar unpleasant procedures should be conducted by women. Those who, for any reason, temperamental or otherwise, feel that pure clinical work is not for them, may well consider the enormous possibilities offered by radiology and electro-therapeutics.^59^

From cancer research to radiography, the appeal of the new radiation techniques was compelling.

In 1925, the MWF’s Cancer Committee invited Dr. Elizabeth Hurdon to become research officer of the project. A graduate of The University of Toronto, English-born Elizabeth Hurdon was well qualified to undertake the work. After studying at the Johns Hopkins Hospital under William Osler, she had taken up a post as associate in gynecology at the Johns Hopkins University and collaborated with Howard Kelly in the publication of two important textbooks.^60^ Most importantly, under the “Big Four” of the Johns Hopkins Hospital Hurdon had gained a wide experience of teamwork. She was thus capable of organizing the scheme along the lines already suggested by Donaldson and others, as an integrated organization managed by a consultant expert.^61^ Once appointed to the position of director, Hurdon became the linchpin of the scheme. The MWF’s project gave participating gynecologists responsibility for carrying out the treatment, but the dosage and technique were determined by the Committee. The Director transported the radium to the four participating hospitals [Other P-157] (the South London Hospital for Women, the Elizabeth Garrett Anderson Hospital, the Royal Free Hospital, and the New Sussex Hospital) in turn, attended the insertion of the radium, advised the dosage, and kept in touch with the patients, following up each case and making records.

The research had the approval of the MRC’s Radiology Committee, and from 1925 onward it was included in its research program. Quite apart from Chambers’s links with Sidney Russ, the view that cancer of the uterus was a “female” concern played a crucial role in securing the Committee’s support. As Walter Fletcher, the MRC’s first secretary, explained in 1925 to George Newman, the chief medical officer to the Ministry of Health, “these women are dealing with cancer in women, and in so far as this radium is concerned, chiefly with cancer of the womb. . . . of all the radium jobs, this seems the most appropriate for women to tackle, for obvious reasons.”^62^ What he feared, however, was that the women’s scheme might become a new “campaign.” Fletcher had opposed the establishment of the British Empire Cancer Campaign (BECC) two years earlier, partly on the grounds that it would divert much-needed funds away from established research bodies; to his mind, the MWF’s project posed similar dangers.

Fletcher’s fears were to prove unfounded. The MWF’s appeal collected very little money, and in the end it was the BECC that funded Hurdon’s salary.^63^ The BECC also loaned the 500 mg of radium that the MWF needed to initiate the project. In order to elaborate the technique to be followed, Hurdon toured radium therapy centers in Europe and America collecting information and comparing methods of treatment. On her return the Committee agreed to adopt the technique developed by James Heyman at Stockholm’s Radiumhemmet, which involved the intracavitary insertion of radium in three separate applications, two weeks apart.^64^ The first patient, an elderly woman with advanced cervical carcinoma, was treated by Louisa Martindale at the New Sussex Hospital in September 1925. In the next three years, more than three hundred patients were treated at the four women’s hospitals concerned. Dr. Hurdon attended every operation.

By 1929, 322 cases had been treated, and of these only 68 were operable. The Cancer Committee had adopted the five-year surgical “cure” as the gold standard of successful treatment; hence, no definite claim could be made about the value of the therapy. The results were said to [Other P-158] be “encouraging,” however. According to Helen Chambers, 90 percent of the operable cases were free from all the signs of cancer, while all the inoperable cases were “materially benefited.” Most of the deaths were attributed to “asthenia due to internal metastases”; only one death had occurred as a direct result of the treatment.^65^ The statistics provided by Chambers also indicated significant changes in treatment patterns at the four participating institutions. Between 1925 and 1927, eighteen cases had been referred for recurrence after operation; by 1928 the number of such cases had dropped to two: “This is accounted for by the fact that most of our women surgeons do not operate at the present time on cases of cervical cancer, but prefer to treat them at a Radium Clinic,” Chambers explained.^66^

The evolution of the clinic into a central hospital closely reflected the Radiology Committee’s growing support for the centralization of radiotherapy in a few specialist institutions. By the late 1920s, advocates of radium therapy had become disillusioned with providing the general hospitals with radium for research purposes. The difficulties highlighted by members of the Radiology Committee included a paucity of beds for cancer research, poor record keeping and follow up, and inefficient use of radium.^67^ Writing in 1930, Helen Chambers claimed that a shortage of beds for treatment, the inefficient use of radium owing to the time lost in transit, and the problem of following up cases in the absence of a central out-patient department were the main factors that had led to the establishment of the Marie Curie Hospital in 1929. “By this time,” she added,

it was generally recognised that Radium therapy was a highly specialised field of work which should only be undertaken at a Centre designed and equipped for the purpose. No one should use Radium who had not had special training. It was realised that the success of the treatment depended entirely upon careful dosage and technique and that the co-ordination of an organised team was essential.^68^

The new hospital was opened in London’s Hampstead district under the direction of Elizabeth Hurdon. It had thirty beds, both public and private; an operating theater; and a pathological laboratory. The medical staff consisted of seventeen surgeons, five physicians, a pathologist, and a radiologist, backed up by a “scientific advisory council” that interestingly included Walter Fletcher and three members of the MRC’s Radiology [Other P-159] Committee: Sidney Russ, Sir Cuthbert Wallace, and Professor E. H. Kettle. Marie Curie was most interested in the project and was pleased to allow her name to be given to the new hospital.^69^

In the previous century, the women-run hospitals had attracted support from titled ladies and upper-class married women philanthropists. In the late 1920s, the list of subscribers to the Marie Curie Hospital still included wealthy philanthropists, but in addition it boasted prominent feminists such as Margaret Bondfield, the Labour MP; Lady Rhondda, proprietor of the liberal feminist paper *Time and Tide*; and Millicent Fawcett, former president of the National Union of Women’s Suffrage and younger sister of Elizabeth Garrett Anderson. Also represented were campaigners for women’s welfare like Eleanor Rathbone, the Family Allowance pioneer, and women doctors themselves; indeed, the largest donation (£10,000) came from Dr. Elizabeth Courtauld, a distant relative of the textiles manufacturer Samuel Courtauld.^70^ The medical women’s cause was championed by the *New Statesman*, the Fabian Socialist weekly founded in 1912 by Sidney and Beatrice Webb. Writing under the pseudonym “Lens,” eugenicist doctor Caleb Saleeby, a Fabian socialist and friend of George Bernard Shaw, hailed the Marie Curie Hospital as a new beginning in cancer therapy. An enthusiast for radium, Saleeby vehemently criticized the “monstrously selfish, arrogant, obstructive and anti-social record of the surgeons in this country as a body in respect of the radiation of cancer,” arguing that “wherever radium is available, the ghastly and deadly operation of panhysterectomy should be condemned as malpraxis.”^71^ Saleeby welcomed the medical women’s plan to extend radium therapy to the treatment of breast cancer, as advocated by surgeon Geoffrey Keynes: “Let us rejoice that, at last, after thousands of years, mankind may begin to say, *Exit* the surgery of cancer.”^72^ Saleeby’s strongly worded articles were condemned by the medical press as “mischievous claptrap,” but they did succeed in raising a neat sum for the new hospital.

The hospital expanded in the 1930s with the addition of an adjoining building in 1933, the provision of apparatus for deep X-ray therapy in 1934, and the establishment in 1937 of a research laboratory equipped to house animals for work in experimental pathology.^73^ As a tribute to Helen Chambers, who had died of breast cancer in 1935, the new [Other P-160] research facilities were named after her. By the outbreak of the Second World War the initial investigation on the use of radium for carcinoma of the cervix had been extended to the treatment of inoperable cancers of the breast. A statistical analysis of cases of uterine cancer treated at the hospital since its foundation showed that the five-year survival rate in the early cases was 83 percent, and 30 percent in the more serious cases. Writing in 1951, Louisa Martindale attributed the good results to the treatment protocol, which included strict asepsis and the application of radium by fully trained surgeons, on patients who were deemed to be fit for the treatment.^74^ In addition, she highlighted both the significance of gender and the importance of teamwork: “The director is always present with her advice and co-operation. Indeed there is much to be said for the Marie Curie Hospital woman surgeon, not only because she is consulted by the patient earlier in her case, when the symptoms are only slight, but because she agrees to follow out a certain technique and is meticulous in its application.”^75^

## Conclusion

Contemporary male gynecologists recognized the key role that cervical cancer had played in the establishment of radiotherapy. Thus Frank Cook, writing in 1954, stated: “To quote Malcolm Donaldson (1933): ‘Gynaecology was the realm in which radium therapy was first used to any great extent; and in this field it still has its greatest value.’ It has more recently been said with a considerable degree of truth that the history of radiotherapy of cervical cancer well represents the history of radiotherapy as a whole.”^76^ Cook singled out the Marie Curie Hospital for special mention, emphasizing the statistical reliability of its results and the benefits conferred to innumerable patients.

Underlying this success story was a unique combination of professional and ideological factors that had served to make radiotherapy for cervical cancer a “woman’s issue.” Medical women’s entry into the field was motivated by long-standing traditions of service to other women, especially those less fortunate than themselves, but it also served as a strategy [Other P-161] for professional advancement at a time when a career structure based around specialist hospital appointments was gradually crystallizing within British medicine.^77^ While die-hard male surgeons sought to preserve the preeminence of operative treatment for cervical cancer, women surgeons found in radiotherapy a means of asserting their commitment to compassionate cancer care.

## Footnotes

1
	    MurphyCaroline“A History of Radiotherapy to 1950: Cancer and Radiotherapy in Britain, 1850–1950” (Ph.D. diss., University of Manchester, 1986), chap. 5 2531 ElstonMary Ann“Run by Women, (Mainly) for Women: Medical Women’s Hospitals in Britain, 1866–1948,”Women and Modern MedicineLawrenceConradHardyAnneAmsterdamRodopi200173107 on p. 90 11603159
	    
	

2
	    WilliamsJohnCancer of the Uterus: Being the Harveian Lectures for 1886LondonLewis18881
	    
	

3
	    PinellPatriceThe Fight against Cancer: France 1890–1940LondonRoutledge20021
	    
	

4
	    WestCharlesLectures on the Diseases of WomenLondonChurchill1858395
	    
	

5
	    McCannFrederick JohnCancer of the Womb: Its Symptoms, Diagnosis, Prognosis, and TreatmentLondonFrowde19078687
	    
	

6
	    CroweSamuel JamesHalsted of Johns Hopkins: The Man and His MenSpringfield, Ill.Thomas1957 LernerBarron H.The Breast Cancer Wars: Fear, Hope and the Pursuit of a Cure in Twentieth-Century AmericaOxfordOxford University Press2003172711949674 . On the development of radical surgery, see BriegerGert H.“From Conservative to Radical Surgery in Late Nineteenth-Century America,”Medical Theory, Surgical Practice: Studies in the History of SurgeryLawrenceChristopherLondonRoutledge199521631
	    
	

7
	    ClarkJohn G.“More Radical Method of Performing Hysterectomy for Cancer of the Uterus,”Johns Hopkins Hosp. Bull18956120 . For an account of the early history of this procedure, see BonneyVictor“Wertheim’s Operation in Retrospect,”Lancet19491637391811730110.1016/s0140-6736(49)91740-7 KellyHowardOperative Gynecology2d ed2New YorkAppleton1909246869
	    
	

8
	    “A Discussion on the Diagnosis and Treatment of Cancer of the Uterus” (leading article)Brit. Med. J.19052689704 . The gynecological profession was sharply divided on the question of lymphadenectomy: many doctors thought that the breast/cervical cancer analogy was wrong. See, e.g., McCannCancer of the Womb5 96
	    
	

9
	    MoscucciOrnellaThe Science of Woman: Gynaecology and Gender in England, 1800–1929CambridgeCambridge University Press1990
	    
	

10
	    McCannCancer of the Womb589
	    
	

11
	    SinclairWilliam Japp“Carcinoma in Women, Chiefly in Its Clinical Aspects,”Brit. Med. J.1902232127 on p. 325
	      
	    
	

12
	    See SpencerHerbert“A Discussion of the Measures to Be Recommended to Secure the Earlier Recognition of Uterine Cancer,”Brit. Med. J.1907243140 DarmonPierreLes cellules folles: L’homme face au cancer de l’Antiquité à nos joursParisLibrairie Plon1993220
	    
	

13
	    ShawWilliam Fletcher“Wertheim’s Hysterectomy for Carcinoma of the Cervix,”Lancet19272538
	    
	

14
	    ChamberlainGeoffreyVictor Bonney: The Gynaecological Surgeon of the Twentieth CenturyNew YorkParthenon2000
	    
	

15
	    Ibid., p. 46.
	

16
	    KellyOperative Gynecology 7 2468
	    
	

17
	    LewersArthur H. N.Cancer of the UterusLondonLewis19021
	    
	

18
	    Murphy“History of Radiotherapy”1chap. 334
	    
	

19
	    Founded in 1896 under the title *Archives of Clinical Skiagraphy* Murphy“History of Radiotherapy”1chap. 210
	    
	

20
	    See, e.g., BashfordErnest F.“Cancer, Credulity and Quackery,”Brit. Med. J.1911112213010.1136/bmj.1.2630.1221PMC233393620765638
	    
	

21
	    SloanS.“Report of the Glasgow Obstetric and Gynaecological Society,”J. Obstet. & Gyn.19045309
	    
	

22
	    FairbairnJ. S.“Röntgen Rays in Obstetrics and Gynaecology,”J. Obstet. & Gyn.1905736768 on p. 368
	      
	    
	

23
	    RouthAmand“Minor Uterine Operations,”A System of GynaecologyAllbuttThomasPlayfairW.2nd ed.LondonMacmillan1906809
	    
	

24
	    PinchA. E. Hayward“A Report of the Work of the Radium Institute,”Brit. Med. J.1913114965 on p. 153 10.1136/bmj.1.2717.149PMC229850620766480
	    
	

25
	    ButlinHenry T.“On Radium in the Treatment of Cancer and Some Associated Conditions,”Lancet19092141114 on p. 1414
	      
	    
	

26
	    BonneyVictor“A Review of Modern Gynaecological Practice,”Lancet19152128389 on p. 1285
	      
	    
	

27
	    “Radium Therapy” (leading article)Brit. Med. J.19201644 45
	    
	

28
	    BarclayA. E.“The New Importance of Radium,”Lancet19291106162 on p. 1061
	      
	    
	

29
	    For a biography of Berkeley, see Sir PeelJohnThe Lives of the Fellows of the Royal College of Obstetricians and Gynaecologists 1929–1969LondonHeinemann19766972
	    
	

30
	    “Radiotherapy in Cancer of the Cervix” (leading article)Brit. Med. J.1933224344
	    
	

31
	    MartindaleLouisaA Woman SurgeonLondonGollancz1951115 . On the reception of the Erlangen method in Britain, see SerwerPaul D.“The Rise of Radiation Protection: Science, Medicine, and Technology in Society, 1896–1935” Ph.D. diss. Princeton University1978
	    
	

32
	    ChambersHelenRussSidney“Principles of Radiological Treatment and Their Bearing on Hospital X-ray Organization,”Brit. Med. J.1935291110.1136/bmj.2.3887.9PMC246099320779208
	    
	

33
	    Septic infections were attributed to previous operative treatment. See “Radiotherapy in Cancer of the Cervix”30 243
	    
	

34
	    MartindaleWoman Surgeon31115
	    
	

35
	    Ibid., p. 114.
	

36
	    “The Radium Institute,”Brit. Med. J.191123024 on p. 302 PMC233204420765757
	    
	

37
	    “The Radium Institute,”Lancet1911239699 on p. 396
	      
	    
	

38
	    See Murphy“History of Radiotherapy”1 CantorDavid“The MRC’s Support for Experimental Radiology during the Inter-War Years,”Historical Perspectives on the Role of the MRC: Essays in the History of the Medical Research Council of the United Kingdom and Its Predecessor, the Medical Research Committee, 1913–1953AustokerJoanBryderLindaOxfordOxford University Press1989181204
	    
	

39
	    Barclay“New Importance of Radium”28 1062
	    
	

40
	    BonneyVictor“Surgical Treatment of Carcinoma of the Cervix,”Lancet1930127782 on p. 282
	      
	    
	

41
	    ShawWilliam Fletcher“Treatment of Cancer by Radium” (letter)*Brit. Med J.*192721244
	    
	

42
	    As radium enthusiasts pointed out, the case for radical hysterectomy also rested largely on foreign statistics. See BirkettG. E.“Treatment of Cancer by Radium” (letter)Brit. Med. J.1928175 . For an account of the surgery vs. radiotherapy debate in France, see PinellFight against Cancer 311523 . The development of radiotherapy services in Canada is discussed by HayterCharles R. R.An Element of Hope: Radium and the Response to Cancer in Canada, 1900–1940MontrealMcGill-Queen’s University Press2005
	    
	

43
	    Quoted in Cantor“MRC’s Support” 38 190
	    
	

44
	    DonaldsonMalcolm“A Suggestion for the Organization of Clinical Research in Cancer,”Brit. Med. J.193316869 on p. 69 10.1136/bmj.1.3758.68PMC236785220777298 On the concept of teamwork in medicine between the two world wars, see SturdySteveCooterRoger“Science, Scientific Management, and the Transformation of Medicine in Britain, c. 1870–1950,”Hist. Sci.1998364216610.1177/00732753980360040211620554
	    
	

45
	    “Radiotherapy in Cancer of the Cervix” 30 243 (emphasis added)
	      
	    
	

46
	    Ibid.
	

47
	    Ibid., p. 244.
	

48
	    “Radium for Cancer” (leading article)Lancet1928197374 on p. 973
	      
	    
	

49
	    Ibid.
	

50
	    
	      See Murphy“History of Radiotherapy” 1chap. 51418
	    
	

51
	    Cantor, “MRC’s Support” (n. 38).
	

52
	    Biographical data on Helen Chambers are drawn from her obituaries: Lancet1935222829 Med. Women’s Fed. Quart. Rev.Jan.19355861 . Chambers’s work on cancer immunity produced meager results, and in MRC circles there was some skepticism about the clinical applicability of the research. See Walter Fletcher to George Newman, 15 December 1923, National Archives (NA), London, FD 1/2037.
	

53
	    ChambersHelen“The Marie Curie Hospital,”Med. Women’s Fed. Newsl.319301923
	    
	

54
	    WorboysMichael“Unsexing Gonorrhoea: Bacteriologists, Gynaecologists, and Suffragists in Britain, 1860–1920,”Soc. Hist. Med.2004174159
	    
	

55
	    See, e.g., PankhurstSylviaSave the MothersLondonKnopf193046
	    
	

56
	    Sackville-WestVitaThe Marie Curie Hospital194720Contemporary Medical Archives Centre (CMAC), Wellcome LibraryLondon SA/MWF/C.41
	      
	    
	

57
	    ScottAnne L.“Physical Purity Feminism and State Medicine in Late Nineteenth-Century England,”Women’s Hist. Rev.199946255222619785 MoscucciScience of Woman 9 12425
	    
	

58
	    Sackville-WestMarie Curie Hospital5620
	    
	

59
	    MagillMary“The Practice of Radiology and Electro-Therapeutics for Medical Women,”Med. Women’s Fed. Newsl.719252126 on p. 21
	      
	    
	

60
	    See Elizabeth Hurdon’s obituaryBrit. Med. J.19412299
	    
	

61
	    Sturdy and Cooter, “Science, Scientific Management” (n. 44).
	

62
	    Fletcher to Newman, 3 April 1925, NA, FD 1/697.
	

63
	    Fletcher to BECC secretary, 4 August 1925, ibid.
	

64
	    The aim was to damage malignant cells by the first dose, and to destroy all cancer cells by the succeeding applications, thus enabling the vascular connective tissue to recover in the intervals. See ElizabethHurdonCancer of the UterusOxfordOxford University Press194253
	    
	

65
	    Chambers, “Marie Curie Hospital” (n. 53), p. 21.
	

66
	    Ibid., p. 22.
	

67
	    See Cantor, “MRC’s Support” (n. 38).
	

68
	    Chambers, “Marie Curie Hospital” (n. 53), p. 20.
	

69
	    “The Marie Curie Hospital,”Lancet1934152728
	    
	

70
	    See NA, FD 1/697 for a list of early supporters.
	

71
	    Lens (pseud.)“The Marie Curie Hospital,”New Statesman31929692
	    
	

72
	    Ibid.
	

73
	    In the light of feminists’ traditional opposition to vivisection, this was an interesting development. It deserves further examination.
	

74
	    MartindaleWoman Surgeon 31 20910 . See also HurdonCancer of the Uterus6467
	    
	

75
	    MartindaleWoman Surgeon 31 20910
	    
	

76
	    CookFrank“The Progress of Radio-Therapy in Gynaecology,”Historical Review of British Obstetrics and Gynaecology 1800–1950Munro KerrJ. M.JohnstoneR. W.PhillipsMiles H.EdinburghLivingstone195438289 on p. 383
	      
	    
	

